# Phytochemical Composition, Bioactive Compounds, and Antidiabetic Potential of Four Medicinal Plants Native to the UAE: *Capparis spinosa, Citrullus colocynthis, Morus alba*, and *Rhazya stricta*

**DOI:** 10.3390/biology14091146

**Published:** 2025-08-30

**Authors:** Seham M. Al Raish, Razan S. Almasri, Alaa S. Bedir, Aya A. Elkahwagy

**Affiliations:** 1 Department of Biology, College of Science, United Arab Emirates University, Al Ain 15551, United Arab Emirates; 2 Department of Nutrition, College of Medicine and Health Science, United Arab Emirates University, Al Ain 15551, United Arab Emirates; 201950110@uaeu.ac.ae (R.S.A.); 201950078@uaeu.ac.ae (A.S.B.); 3 Department of Pharmacy Practice, Faculty of Pharmacy, Sinai University—Arish Branch, Arish 45511, Egypt; su1301258@su.edu.eg

**Keywords:** medicinal plants, antidiabetic, metabolic disorders, phytochemicals, *Capparis spinosa*, Citrullus colocynthis, Morus alba, Rhazya stricta, sustainable healthcare, bioactive metabolites

## Abstract

This review highlights the significant therapeutic properties of four indigenous plants in the United Arab Emirates. These include *Capparis spinosa L.* (family: Capparaceae), commonly known as caper and locally referred to as Kabir; *Citrullus colocynthis (L) Schrad.* (family: Cucurbitaceae), known in English as bitter apple and locally as Alhanzal; *Morus alba L.* (family: Moraceae), referred to as white mulberry and locally named Firsad; and *Rhazya stricta Decne.* (family: Apocynaceae), commonly called harmal-e-shami and known locally as Alhi-rimi. These species are traditionally used for various ethnobotanical purposes and are important components of the region’s flora, such as managing diabetes and associated metabolic disorders. These plants contain diverse bioactive compounds with notable pharmacological activities. For example, caper exhibits antidiabetic effects through flavonoids such as quercetin and kaempferol, which enhance insulin sensitivity and lower blood glucose levels. Bitter apple is rich in cucurbitacins and alkaloids that lower glycated hemoglobin and support pancreatic β-cell function. White mulberry contains chlorogenic acid, rutin, and 1-deoxynojirimycin, which improve glucose uptake, inhibit α-glucosidase, and reduce oxidative stress. Harmal-e-shami exhibits variable antidiabetic activity, including dipeptidyl peptidase-IV inhibition and enhancement of glucagon-like peptide-1 secretion, which is influenced by the type and dosage of the extract. Despite these promising effects, challenges remain in standardization, phytochemical variability, and clinical validation. This review underscores the therapeutic potential of these plants and recommends further research for their integration into sustainable, plant-based diabetes management strategies.

## Simple Summary

This review addresses the increasing burden of diabetes and metabolic disorders by exploring the therapeutic potential of four medicinal plants native to the United Arab Emirates: *Capparis spinosa* (Kabir) (caper), *Citrullus colocynthis* (Alhanzal) (bitter apple), *Morus alba* (Firsad) (white mulberry), and *Rhazya stricta* (Alhi-riml or harmal-e-shami). The primary objective was to consolidate current scientific evidence on the phytochemical constituents and antidiabetic properties of these plants. Caper exhibits glucose-lowering effects through flavonoids, enhancing insulin sensitivity. Bitter apples contain cucurbitacins that significantly reduce blood glucose and glycated hemoglobin levels. White mulberry, rich in chlorogenic acid and 1-deoxynojirimycin, effectively controls postprandial glucose and reduces oxidative stress. Harmal-e-shami exhibits variable antidiabetic activity through the inhibition of dipeptidyl peptidase-IV and the enhancement of glucagon-like peptide-1 secretion. Despite these promising findings, challenges remain in standardizing preparations and clinical validation. This study emphasizes the potential integration of these plants into sustainable diabetes management strategies, highlighting their relevance in modern healthcare and recommending further research for clinical applications.

## 1. Introduction

Diabetes mellitus (DM) is one of the most significant and rapidly growing chronic diseases worldwide, affecting an estimated 537 million adults in 2021, with projections reaching 783 million by 2045 [1,2,3,4,5]. In the United Arab Emirates (UAE), recent epidemiological surveys indicate prevalence rates exceeding 16%, among the highest globally, driven by rapid urbanization, sedentary lifestyles, and dietary shifts [1,2,3,4,5]. The economic and health impact is substantial, as DM is a leading cause of cardiovascular disease, nephropathy, neuropathy, and retinopathy [1,2,3,4,5]. Although conventional pharmacotherapies—such as insulin and oral hypoglycemic agents—remain the standard for glycemic control [6,7], their long-term use is often limited by adverse effects, high cost, and suboptimal prevention of chronic complications [6,7]. These limitations have intensified interest in complementary strategies that are safer, more affordable, and culturally relevant [8,9].

Medicinal plants (MPs) have been integral to human healthcare systems for millennia, forming the basis of both traditional and modern therapeutics [8,9]. They are rich in structurally diverse bioactive compounds—flavonoids, alkaloids, terpenoids, saponins, and phenolic acids—that confer a wide range of pharmacological effects, including antidiabetic, antioxidant, anti-inflammatory, antimicrobial, cardioprotective, neuroprotective, and immunomodulatory properties [10,11,12,13]. Over the past two decades, ethnopharmacological investigations in the UAE have documented more than 100 species with recognized medicinal applications, adapted to extreme arid conditions and often exhibiting elevated phytochemical content due to environmental stress (Comprehensive Ethnopharmacological Analysis of Medicinal Plants in the UAE: *Lawsonia inermis*, *Nigella sativa*, *Ziziphus spina-christi*, *Allium cepa*, *Allium sativum*, *Cymbopogon schoenanthus*, *Matricaria aurea*, *Phoenix dactylifera*, *Portulaca oleracea*, *Reichardia tingitana*, *Salvadora persica*, *Solanum lycopersicum*, *Trigonella foenum-graecum*, *Withania somnifera*, and *Ziziphus lotus*) [14,15,16].

The therapeutic relevance of MPs extends beyond metabolic disorders. In dermatology, plant-based formulations have shown promise in promoting hair growth and managing alopecia through mechanisms such as enhancing microcirculation, modulating inflammatory pathways, and reducing oxidative stress (Alopecia and the Role of Herbal Medicine in Hair Growth Promotion: Mechanisms, Efficacy, and Safety). These multi-target actions align with the principles of integrative medicine, which favor interventions capable of addressing interconnected pathological processes [17].

Several MPs traditionally used in the UAE have been validated for antidiabetic activity through in vitro, in vivo, and limited clinical studies. *Capparis spinosa* has demonstrated α-glucosidase inhibitory activity, insulin-sensitizing effects, and reductions in oxidative stress markers [1,4,5,18,19,20,21,22]. *Citrullus colocynthis* fruit extracts have shown significant hypoglycemic effects, improvements in lipid profiles, and potential pancreatic β-cell protection [1,4,5,18,19,20,21,22]. *Morus alba* leaf flavonoids modulate carbohydrate-digesting enzymes, improve postprandial glucose levels, and exert antioxidant activity [1,4,5,18,19,20,21,22]. *Rhazya stricta* alkaloids and flavonoids exhibit potent antioxidant properties, enhance glucose uptake, and may protect pancreatic β-cells from oxidative damage [1,4,5,18,19,20,21,22]. Additionally, globally recognized species such as Allium sativum, Nigella sativa, and Withania somnifera cultivated or utilized within the UAE have been associated with improved glycemic control, cardiovascular protection, and immune modulation [11,12]. Phytochemical yields in these plants can be notably high under UAE cultivation; for example, elevated phenolic concentrations in *Morus alba* and enhanced alkaloid profiles in *Rhazya stricta* potentially increase their therapeutic potency.

This review focuses on four medicinal plants *Capparis spinosa*, *Citrullus colocynthis*, *Morus alba*, and *Rhazya stricta* selected for their ethnomedicinal importance in the UAE, confirmed botanical presence, and availability of robust pharmacological data [1,4,5,18,19,20,21,22]. By integrating ethnobotanical heritage with contemporary biomedical research, this work aims to critically evaluate their therapeutic potential in DM and metabolic syndrome, identify gaps in mechanistic and clinical evidence, and propose strategies for sustainable, culturally attuned clinical applications.

## 2. Materials and Methods

### 2.1. Literature Search

A comprehensive literature search was performed using Web of Science, Scopus, PubMed, and Google Scholar to find relevant studies published between 1998 and 2025. The search included combinations of keywords such as “*Capparis spinosa*,” “*Citrullus colocynthis*,” “*Morus alba*,” “*Rhazya stricta*,” “diabetes,” “antidiabetic,” “phytochemicals,” “bioactive compounds,” and “UAE medicinal plants.” Boolean operators (AND/OR) were used to narrow down the results. Additionally, the reference lists of selected articles were manually screened to find more eligible studies.

### 2.2. Study Selection

Studies were included if they focused on at least one of the selected medicinal plants and provided original data on their antidiabetic or metabolic effects. Eligible studies also reported phytochemical profiles or mechanisms of action and were published in English in peer-reviewed journals. Studies were excluded if they lacked experimental or clinical relevance, addressed unrelated health outcomes, were non-English without translation, or consisted of narrative reviews without original research data.

### 2.3. Data Extraction

Data from the included studies were extracted based on plant species, bioactive constituents, mechanisms of antidiabetic activity, and observed outcomes. Information related to study design, dosage, and safety was also recorded. The findings were synthesized thematically and organized according to the plant species selected. A narrative approach was applied to analyze and present the collective evidence.

## 3. Phytochemical Profiles of the Selected Plants

### 3.1. Capparis spinosa

Caper is a versatile perennial plant that is native to the UAE, thriving in mountainous areas, rocky terrains, limestone formations, wadis, and roadsides, with a flowering period from February to April [23]. Renowned for its health-promoting properties and diverse applications in the food industry, the caper has been extensively studied for its phytochemical profile and biological activities. It contains a rich array of bioactive compounds, including flavonoids, phenolic acids, alkaloids, volatile oils, fatty acids, and polysaccharides [19,24]. These compounds are responsible for its antioxidant, antidiabetic, anticancer, hepatoprotective, neuroprotective, anti-inflammatory, anti-arthritic, and antimicrobial effects [18,20]. Additionally, caper plays a significant role in traditional medicine, where it is used to treat numerous diseases because of its therapeutic potential [25].

The nutraceutical value of caper has been highlighted through its potential as a natural antioxidant and preservative in food systems. Research has demonstrated that its bioactive compounds enhance food preservation, packaging, and safety, making it a valuable addition to innovative food technologies [19,26]. For instance, fermented caper buds and berries exhibit high phenolic content and antioxidant activity, with dry-salted fermentation being more effective in preserving these qualities than brining methods [27]. Moreover, studies on capers from Pantelleria Island emphasize their exceptional nutraceutical relevance, showing strong correlations between their bioactive composition and health benefits, such as antiradical and glyoxal trapping activities [26].

Furthermore, a comparative metabolomic study of wild and cultivated caper from Sardinia reveals significant qualitative and quantitative differences in the bioactive compounds across plant parts, including buds, flowers, and leaves. The findings reveal high levels of glucosinolates, with glucocapparin and rutin as prominent compounds that contribute to their anticarcinogenic and antioxidant properties [28]. These findings underline the agricultural and pharmacological importance of caper and its potential for drug discovery and therapeutic applications.

Quantitative studies have reported that the total flavonoid content in caper fruit extracts ranges from 10.3 to 14.6 mg quercetin equivalents (QE)/g. In contrast, the total phenolic content ranges from 22.4 to 34.9 mg gallic acid equivalents (GAE)/g, depending on the extraction method and geographic origin [5,19]. In preclinical research, administration of 20 mg/kg aqueous extract significantly lowered fasting blood glucose in diabetic rats [29]. A clinical trial administering 400 mg/day of fruit extract for eight weeks showed notable reductions in fasting blood glucose and HbA1c levels in patients with type 2 diabetes mellitus (T2DM), without adverse hepatic or renal effects [30]. Additionally, fermented buds have been shown to contain high levels of rutin (up to 9.8 mg/g DW), further supporting their nutraceutical value [4]. These findings affirm the pharmacological relevance of caper, particularly in metabolic and oxidative stress-related disorders.

**Figure 1:**
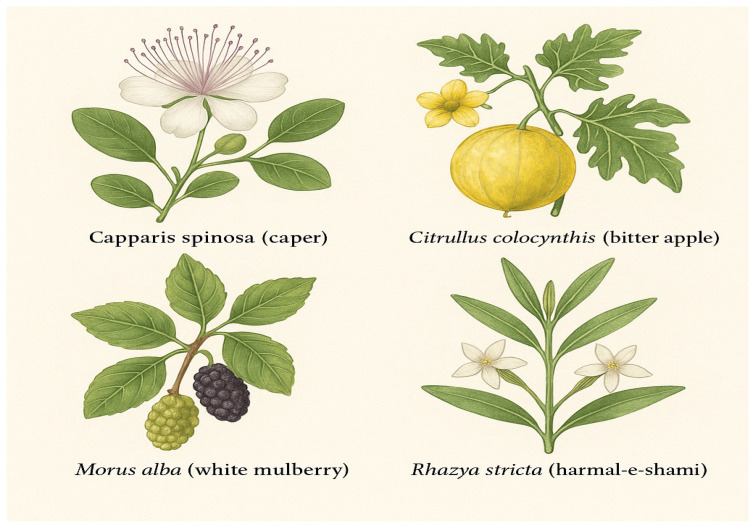
Representative illustrations of the four medicinal plants native to the UAE: caper, bitter apple, white mulberry, and harmal-e-shami.

In summary, caper stands out as a plant of remarkable nutritional, medicinal, and industrial significance. Its multifaceted benefits, including its functional properties in food preservation and its array of health-promoting activities, make it an excellent candidate for ongoing scientific research and commercial development (Figure 1).

### 3.2. Citrullus colocynthis

Bitter apple is a valuable perennial plant that is widely distributed in desert regions; it is native to the UAE, where it thrives in sandy and gravelly soils, flowering from May to October [23,31]. Recognized for its broad spectrum of traditional medicinal uses, bitter apple has been employed to treat diabetes, respiratory conditions, gastrointestinal disorders, and various infections [32,33]. The plant’s fruit contains a rich array of bioactive compounds, including glycosides, flavonoids, alkaloids, fatty acids, and essential oils, with significant amounts of cucurbitacins and colocynthosides identified [21,32,34,35,36].

Recent research has highlighted the diverse biological activities of bitter apple, which include antioxidant, cytotoxic, antidiabetic, antilipidemic, insecticidal, antimicrobial, and anti-inflammatory properties [21,32,37,38]. For instance, a study employed TLC-bioautography-MS techniques to identify antioxidant and antidiabetic compounds in bitter apple, demonstrating its potential for high-throughput screening of bioactive molecules [39]. Additionally, another study explored its cytotoxic effects, showing that crude alcoholic extracts of the fruit significantly inhibit the growth of human hepatocyte carcinoma (Hep-G2) cells. The highest inhibition rate, observed at 20 µg/mL, reached 93.36% after 72 h, underscoring the plant’s promise in cancer treatment [40].

Apart from its pharmacological significance, the bitter apple holds substantial nutritional value. Its seeds are rich in protein, essential minerals, and edible oils, hence making it a necessary resource for nutraceutical development [41]. The synergistic effects of its bioactive compounds, coupled with minimal adverse effects, make this plant a candidate for the pharmaceutical and functional food industries.

Phytochemical analyses of bitter apple fruit have identified cucurbitacin E as a major triterpenoid, with concentrations reaching up to 3.7 mg/g in methanolic extracts [38]. Total alkaloid content in fruit pulp has been quantified at 2.6–5.4% dry weight, contributing to its biological activity [21,37]. In vivo studies have demonstrated that ethanolic extracts (100–200 mg/kg) significantly reduce blood glucose levels in diabetic rat models within 14 days [42]. In clinical settings, administration of bitter apple fruit powder at 300 mg/day for 2 months resulted in a 21.6% decrease in HbA1c and improved lipid profiles in type 2 diabetic patients. However, higher doses (>500 mg) have been associated with gastrointestinal irritation, underscoring the importance of standardizing the dose [43].

Bitter apple offers immense potential for medicinal, nutritional, and industrial applications. Its bioactive compound profile, traditional uses, and recent scientific evidence establish it as a promising candidate for further research and commercial exploitation in addressing various health and environmental challenges (Figure 1).

### 3.3. Morus alba

White mulberries are a versatile perennial tree. Renowned for its traditional and medicinal uses, white mulberry has been extensively cultivated, including on private farms in the UAE. It thrives in the UAE’s climate, flowering between March and June [23]. Various parts of the plant, including leaves, fruits, branches, and roots, have been utilized in traditional medicine to treat ailments such as rheumatism, diabetes, and hypertension, as well as to support functions like improving eyesight, strengthening joints, and facilitating urine discharge [44,45].

Phytochemical studies reveal that white mulberry contains a diverse array of bioactive compounds, including tannins, steroids, alkaloids, flavonoids, anthocyanins, phenolic acids, and stilbenoids, which are responsible for its wide-ranging pharmacological properties [44,46]. The leaves are particularly noted for their hypoglycemic, anti-inflammatory, and anti-atherosclerotic properties, while the fruits exhibit antioxidant, neuroprotective, and antitumor effects [47,48]. In traditional Asian medicine, mulberry leaves are highly valued for their role in regulating blood glucose and metabolic diseases, supported by modern pharmacological research that demonstrates their antidiabetic and antihyperlipidemic effects [4].

Modern scientific investigations corroborate the traditional medicinal uses of white mulberry and further highlight its potential in treating various health conditions. The plant has demonstrated antimicrobial, anticancer, cardioprotective, and immunomodulatory effects [29,49,50]. Clinical studies have particularly focused on the role of mulberry extracts in reducing blood glucose and cholesterol levels, as well as improving cognitive function [46]. Furthermore, its comprehensive nutritional composition, which includes essential macronutrients, vitamins, and minerals, underscores its potential as a valuable functional food ingredient [47]. The therapeutic and nutritional properties of white mulberry underscore its multifaceted value in traditional medicine, pharmacology, and the development of functional foods. Future research should focus on the bioactive compounds, scaling up production for industrial applications, and integrating their derivatives into modern therapeutic and dietary practices.

Quantitative profiling of white mulberry leaves revealed high levels of DNJ, ranging from 0.11 to 0.36 mg/g DW, responsible for its α-glucosidase inhibitory activity [30]. Total phenolic content in leaf extracts varies from 35.6 to 62.1 mg GAE/g, while flavonoid content ranges from 8.3 to 17.2 mg QE/g, depending on geographic origin and maturity stage [29]. In preclinical trials, supplementation with 200 mg/kg mulberry leaf extract reduced blood glucose by 32–40% in streptozotocin-induced diabetic rats [38]. Clinical studies have confirmed that the ingestion of mulberry leaf extract (1.2 g/day) for 3 months resulted in a significant reduction in postprandial glucose and insulin levels in patients with impaired glucose tolerance [22] (Figure 1).

### 3.4. Rhazya stricta

Rhazya, or harmal-e-shami, is a perennial shrub. In some regions, it is also referred to as Desert Milkweed or Aqeeq (in Arabic-speaking areas). It is a medicinal plant widely found in arid regions, particularly in the Middle East and South Asia. It is native to the UAE [51,52]. It thrives in sandy, gravelly soils, rocky terrains, and wadi beds, flowering from February to June [23]. Traditionally, harmal-e-shami has played a significant role in indigenous medicine systems, being used to treat a wide array of conditions, including diabetes mellitus, syphilis, parasitic infections, rheumatism, hyperglycemia, and the common cold [30,51].

Phytochemical studies have identified glycosides, alkaloids, tannins, and triterpenes as the primary active compounds in harmal-e-shami leaf extracts. These compounds exhibit promising pharmacological activities, including antidiabetic, anticancer, anti-inflammatory, and antioxidant properties [52,53]. Variations in the concentrations of secondary metabolites, such as flavonoids and phenolic compounds, have been observed in response to environmental changes, indicating the plant’s ability to adapt to diverse climatic conditions. Moreover, alkaloids extracted from harmal-e-shami have been reported to possess potential anticancer properties, underscoring the plant’s significance in pharmacological research [53].

Modern scientific investigations continue to corroborate the traditional medicinal uses of harmal-e-shami. A study evaluated its antidiabetic potential through phytochemical profiling of root extracts, revealing its efficacy in lowering blood glucose levels [30]. The medicinal and pharmacological value of harmal-e-shami makes it a significant plant for further research and development in the fields of natural medicine and drug discovery. Its phytochemical diversity and therapeutic potential offer promising avenues for treating various diseases, including chronic and metabolic disorders (Table 1).

Quantitative assessments show that harmal-e-shami leaf extracts contain alkaloids at concentrations up to 3.8% dry weight, including compounds such as rhazinilam and rhazimine [54]. In animal studies, administration of methanolic extracts at 250 mg/kg/day resulted in a significant decrease in blood glucose levels and an improved insulin response in diabetic mice over 21 days [55]. Another study further reported that ethyl acetate fractions of harmal-e-shami reduced plasma glucose by 30.5% and increased insulin by 24.1% compared to untreated diabetic controls [30]. The extract also inhibited dipeptidyl peptidase-IV (DPP-IV) activity by up to 67%, suggesting a multifactorial antidiabetic mechanism [56]. While promising, these effects appear to be dose-dependent and may vary depending on the purity and composition of the extract (Figure 1) (Table 1).

**Table 1:** Medicinal plants in the UAE: phytochemical profiles, traditional uses, and pharmacological potential of caper, bitter apple, white mulberry, and harmal-e-shami.

**Plant Name**	**Family**	**Native Habitat**	**Flowering Period**	**Traditional Uses**	**Bioactive Compounds**	**Pharmacological Properties**	**Key References**
Caper	Capparaceae	UAE, mountains, rocky terrains, limestone formations, wadis, and roadsides	February to April	Treating numerous diseases, including diabetes, microbial infections, inflammation, and liver disorders	Flavonoids (quercetin and kaempferol), glucocapparin, rutin, alkaloids, phenolic acids, and volatile oils	Antioxidant, antidiabetic, hepatoprotective, anti-inflammatory, and organ-protective	[18,19,20,24,57]
Bitter apple	Cucurbitaceae	UAE, desert regions, sandy and gravelly soils	May to October	Treating diabetes, respiratory conditions, gastrointestinal disorders, and infections	Cucurbitacin E, colocynthoside A, flavonoids, alkaloids, glycosides, and phenolic acids	Hypoglycemic, β-cell regenerative, antioxidant, and lipid-lowering	[21,32,33]
White mulberry	Moraceae	UAE, private farms	March to June	Treating rheumatism, diabetes, and hypertension; improving eyesight; strengthening joints	DNJ, chlorogenic acid, rutin, isoquercitrin, gentisic acid, flavonoids, and stilbenoids	α-glucosidase inhibition, insulin sensitizer, antioxidant, anti-inflammatory, and hypoglycemic	[22,29,44,45,58,59]
Harmal-e-shami	Apocynaceae	UAE, sandy gravelly soils, rocky terrains, wadi beds	February to June	Treating diabetes mellitus, syphilis, parasitic infections, hyperglycemia, rheumatism, and fever	Rhazimine, rhazinilam, alkaloids, triterpenes, glycosides, and flavonoids	DPP-IV inhibition, GLP-1 enhancement, insulin sensitivity, hypoglycemic, and antihyperlipidemic	[30,51,52,53,60]

DNJ: 1-deoxynojirimycin; DPP-IV: dipeptidyl peptidase-IV; GLP-1: glucagon-like peptide-1.

## 4. Overview of Antidiabetic Properties of Medicinal Plants in the UAE

### 4.1. Capparis spinosa

Caper is a plant recognized for its diverse phytochemical composition and has been extensively studied for its therapeutic potential, particularly in the management of diabetes. The structure-activity relationship (SAR) of its bioactive compounds suggests that specific hydroxyl group configurations play a critical role in their antidiabetic effects. Notably, quercetin, myricetin, and kaempferol contain a hydroxyl group at position 4′, while quercetin, isorhamnetin, and myricetin possess an additional hydroxyl group at position 3′. These structural features are considered essential for their biological efficacy in regulating glucose and managing diabetes [24].

In vivo studies have substantiated the antidiabetic potential of caper. A study demonstrated that administering 20 mg/kg of aqueous fruit extract significantly reduced fasting blood glucose levels in streptozotocin-induced diabetic rats [61]. Another study further revealed that aqueous extracts of caper decreased endogenous glucose production and improved insulin sensitivity in diabetic mice [11]. Similarly, the root extracts improved glucose levels, lipid profiles, and liver enzyme markers in diabetic rats, suggesting insulin-independent mechanisms [62].

The therapeutic potential of caper in managing T2DM has been increasingly supported by both preclinical and clinical evidence. In a well-characterized animal model of T2DM induced by a high-fat diet and low-dose streptozotocin, oral administration of caper fruit extract at doses of 200 mg/kg and 400 mg/kg for 28 days significantly improved glucose tolerance and reduced fasting blood glucose levels. These effects were accompanied by enhanced antioxidant enzyme activity and preservation of pancreatic histological architecture. Notably, the antidiabetic efficacy observed was comparable to that of metformin administered at 50 mg/kg, suggesting a mechanistic overlap involving oxidative stress modulation and β-cell protection [18].

Clinical findings corroborate the preclinical data. In a randomized, double-blind, placebo-controlled trial, daily supplementation with 400 mg of caper fruit extract for eight weeks in patients with T2DM resulted in statistically significant reductions in both fasting blood glucose (*p* = 0.037) and HbA1c levels (*p* = 0.043). Importantly, the intervention was well tolerated, with no reported adverse effects on hepatic or renal function, supporting the extract’s safety profile and clinical applicability [57].

Further supporting its metabolic benefits, another randomized controlled study demonstrated that caper oxymel, a traditional preparation, effectively slowed the progression of hyperglycemia and contributed to a significant reduction in body mass index among diabetic individuals [1].

Collectively, these findings underscore the potential of caper as a safe and effective natural adjunct for glycemic control and metabolic regulation in T2DM. However, larger-scale and longer-term clinical trials are warranted to validate these outcomes and to explore the underlying mechanisms in human subjects.

In summary, caper exhibits strong antidiabetic potential supported by both preclinical and clinical evidence. Its effects are primarily mediated by flavonoids that enhance insulin sensitivity, reduce blood glucose, and protect against oxidative stress, making it a promising candidate for future therapeutic applications.

### 4.2. Citrullus colocynthis

Bitter apple contains a diverse array of bioactive compounds that contribute to its therapeutic potential, particularly in diabetes management. Studies systematically reported the presence of cucurbitacins (triterpenoids and their glycosides) in this species, including cucurbitacins A–L and cucurbitacin E 2-O-β-D-glucopyranoside [38,63]. Notably, cucurbitacin E is the primary component in bitter apple fruit pulp [64]. Preliminary phytochemical screening has also revealed the presence of alkaloids, flavonoids, and phenolic acids. Twelve alkaloids, including various quinoline derivatives, nicotinamide, and uracil, have been detected in bitter apple fruits [21,37,65]. The fruit extracts have demonstrated insulin-enhancing activity, and bitter apple has been shown to directly reduce the formation of glycated hemoglobin (HbA1c) [20,21,66]. These diverse bioactive compounds and their mechanisms of action highlight the potential of bitter apple as a natural therapeutic agent for managing diabetes and its associated complications. Research evidence strongly supports the antidiabetic potential of bitter apples. Studies reported a concentration–response correlation between fruit extracts and the modulation of insulin secretory response to D-glucose [21,67]. The plant extract exhibited a time-dependent decrease in blood glucose levels, with bitter apple seeds displaying a direct effect on pancreatic beta cells [42,68]. The fruit has shown positive effects in treating diabetic neuropathy and protecting against cognitive impairments, pancreatic β-cell mass, liver/kidney function, and diabetic neuropathic pain [69,70,71,72,73]. Clinical studies have demonstrated the systemic therapeutic effects of bitter apple on T2DM patients, with significant reductions in insulin secretion, blood glucose levels, and glycosylated hemoglobin [43,74,75]. A study showed that bitter apple reduced HDL, triglycerides, cholesterol, and glucose levels by 5%, 6%, 6%, and 35%, respectively, in T2DM patients [21]. These findings collectively underscore the potential of bitter apple as a natural therapeutic agent for managing diabetes and its associated complications.

Bitter apple exhibits potent glucose-lowering and β-cell protective effects attributed to its content of cucurbitacins, alkaloids, and flavonoids. A study demonstrated that aqueous and ethanolic extracts of bitter apple seeds significantly reduced blood glucose levels and improved insulin responsiveness in streptozotocin-induced diabetic rats [67]. Another study further demonstrated that bitter apple extract inhibited the formation of glycated hemoglobin (HbA1c), indicating its potential role in mitigating long-term glycemic damage [66]. A randomized clinical trial reported that 300 mg/day of fruit powder over two months resulted in a significant reduction in fasting glucose and HbA1c levels in type 2 diabetic patients, without observed hepatic or renal toxicity [43].

In conclusion, bitter apple has demonstrated effective glucose-lowering, β-cell regenerative, and anti-inflammatory properties. Its bioactive compounds, particularly cucurbitacins and alkaloids, contribute to its potential role in diabetes management, though dosage safety remains a key consideration.

### 4.3. Morus alba

White mulberry is widely recognized for its diverse phytochemical profile, comprising bioactive compounds with significant pharmacological properties, particularly in the management of diabetes and related metabolic disorders. Among its key bioactive constituents, chlorogenic acid, a phenolic compound from the hydroxycinnamic family, is known for its antioxidant and antidiabetic properties [29,76]. Another important phenolic compound, gentisic acid, a diphenolic derivative of benzoic acid, has been identified in white mulberry fruit, contributing to its therapeutic potential [29,77]. Additionally, DNJ, an alkaloid predominantly found in white mulberry leaves, plays a crucial role in inhibiting intestinal α-glucosidase, thereby aiding glucose metabolism and exhibiting strong hypoglycemic effects [29].

Flavonoids such as rutin and quercetin-3-O-β-d-glucoside (Q3G) further enhance the antidiabetic potential of white mulberry due to their dual antidiabetic and anti-obesity effects. These flavonoids regulate glucose uptake by activating the Akt and AMP-activated protein kinase (AMPK) signaling pathways while modulating lipid accumulation in adipocytes [45,78]. Additionally, rutin and chlorogenic acid contribute to reducing oxidative stress and mitigating diabetic complications, as demonstrated in both in vitro and in vivo studies [29,76].

Extensive research has identified multiple mechanisms through which white mulberry exerts its antidiabetic effects. Early investigations recognized DNJ and phenolic acids, including chlorogenic acid, as primary contributors to its hypoglycemic activity [58]. This review study went through 29 studies. It was reported that white mulberry leaf extracts not only enhance glucose uptake and modulate insulin secretion but also exhibit antioxidant and anti-inflammatory properties, making them effective in managing diabetic nephropathy [50]. Additionally, compounds such as isoquercitrin and DNJ have been shown to enhance glucose uptake and inhibit advanced glycation end-products (AGEs)-induced cellular damage, further supporting their role in diabetes management [45].

Recent clinical studies have reinforced these findings. A systematic review concluded that white mulberry significantly reduces postprandial glucose and insulin levels, although further rigorous trials are necessary to confirm its long-term efficacy [22]. Similarly, mulberry leaves improve glucose metabolism and mitigate hyperglycemia-induced organ damage by influencing gut microbiota, reducing inflammation, and alleviating oxidative stress [59]. A meta-analysis confirmed significant reductions in postprandial glucose levels following the consumption of white mulberry, underscoring its potential as a natural intervention for blood sugar management [79].

Moreover, Sangzhi alkaloids (SZ-A), derived from white mulberry twigs, have been shown to promote insulin secretion, protect β-cell function, and prevent β-cell dedifferentiation and apoptosis, making them a promising target for diabetes treatment [80]. Collectively, these findings establish white mulberry as a potent natural source of therapeutic agents for diabetes and obesity-related disorders. Through its diverse composition of phenolic acids, flavonoids, and alkaloids, white mulberry demonstrates significant antidiabetic activity by inhibiting α-glucosidase, enhancing insulin sensitivity, reducing oxidative stress, and modulating glucose metabolism. Clinical findings further support its role as a natural intervention for diabetes management, although continued research is necessary to validate its long-term efficacy and underlying mechanisms.

White mulberry is recognized for its hypoglycemic effects, mainly due to bioactive compounds such as DNJ, chlorogenic acid, and rutin. A systematic review and meta-analysis confirmed that white mulberry leaf extract significantly reduces postprandial glucose and insulin levels, with additional benefits on lipid profiles and oxidative stress markers in clinical studies [22]. These outcomes support the plant’s role in modulating carbohydrate metabolism and improving glycemic control. Furthermore, another study demonstrated that chlorogenic acid and rutin are key contributors to the in vivo antidiabetic activity of white mulberry, with observed improvements in blood glucose and insulin sensitivity in diabetic rat models. Together, these studies confirm the therapeutic relevance of white mulberry in the management of metabolic disorders [58].

Overall, white mulberry exhibits a well-documented antidiabetic profile through mechanisms such as α-glucosidase inhibition, insulin sensitization, and reduction of oxidative stress. Its clinical efficacy and favorable safety data support its use as a functional food or complementary therapy.

### 4.4. Rhazya stricta

Harmal-e-shami has been extensively investigated for its potential role in diabetes management, with studies yielding variable outcomes regarding its effects on glucose homeostasis and insulin regulation in animal models. Some research indicates that harmal-e-shami extracts do not produce significant changes in glucose concentrations or other measures of glucose homeostasis in diabetic rats [30,51]. However, other studies report substantial reductions in plasma glucose levels accompanied by increased insulin concentrations following both acute and chronic administration of harmal-e-shami extracts [51,60]. These discrepancies suggest that several factors, including the method of extract preparation, dosage, and duration of administration, may influence the antidiabetic efficacy of harmal-e-shami. Thus, standardization remains a key challenge for future research. Further investigation is required to elucidate the specific bioactive compounds responsible for these effects and to determine the optimal conditions for maximizing their therapeutic potential in diabetes management.

The hypoglycemic activity of harmal-e-shami is further substantiated by its ability to inhibit key enzymes involved in hyperglycemia, including dipeptidyl peptidase-IV (DPP-IV) and β-secretase. Additionally, harmal-e-shami has been shown to enhance glucagon-like peptide-1 (GLP-1) secretion, a critical regulator of glucose metabolism [30,51]. Another notable mechanism through which harmal-e-shami contributes to diabetes management is its influence on adiponectin levels, a hormone closely associated with improved insulin sensitivity, further emphasizing its potential therapeutic role in glycemic control [51,54].

Among the different extract fractions, the ethyl acetate fraction (EF) of harmal-e-shami has demonstrated auspicious glucose-lowering effects, exhibiting efficacy comparable to that of standard antidiabetic drugs [51]. A study attributed these effects to the synergistic action of various phytochemicals, including alkaloids and heterocyclic compounds, present in the plant [30]. Additionally, the methanolic extract of harmal-e-shami leaves significantly reduced blood glucose, cholesterol, and triglyceride levels in streptozotocin-induced hyperglycemic mice. Interestingly, this study found that the hypoglycemic effects were more pronounced in female mice compared to males, suggesting a potential influence of sex on the plant’s efficacy [81].

These findings collectively reinforce the antidiabetic potential of harmal-e-shami as a natural therapeutic agent. Its ability to modulate glucose and lipid metabolism, combined with its enzyme-inhibitory and hormone-regulating properties, underscores its promise for further exploration in diabetes treatment. Future research should focus on the isolation and purification of active compounds, as well as their clinical evaluation, to fully elucidate the therapeutic potential of harmal-e-shami in diabetes management.

Harmal-e-shami exhibits antidiabetic effects through enzyme inhibition and hormonal regulation. A study on root extract fractions showed DPP-IV inhibition of up to 61%, β-secretase inhibition of up to 83%, and elevated GLP-1 secretion, which led to significantly reduced glucose and HbA1c levels in diabetic mice [30]. The ethyl acetate fraction was the most effective. Additionally, methanolic leaf extract lowered fasting glucose, cholesterol, and triglyceride levels, with more pronounced effects in female mice [60]. GC–MS analysis confirmed the presence of active alkaloids, likely contributing to these effects [52].

In summary, harmal-e-shami shows multifaceted antidiabetic activity, including DPP-IV inhibition, GLP-1 enhancement, and adiponectin modulation. While findings are promising, further standardized and clinical studies are needed to validate its therapeutic applications (Table 2).

## 5. Additional Pharmacological Activities of the Reviewed Medicinal Plants

### 5.1. Capparis spinosa

Beyond its antidiabetic effects, caper has demonstrated notable anticancer activity, particularly through flavonoids like rutin and kaempferol, which induce apoptosis and inhibit tumor growth in vitro [25,82,83]. Its anti-inflammatory properties are attributed to the suppression of the NF-κB and COX-2 pathways, with studies showing the inhibition of pro-inflammatory cytokines in lipopolysaccharide-stimulated macrophages [20,83,84,85]. Extracts also exhibit strong antimicrobial activity against both Gram-positive and Gram-negative bacteria, with MIC values as low as 125 µg/mL for *S. aureus* and *E. coli* [19,86]. Cardioprotective effects have also been observed, with reductions in systolic blood pressure and LDL cholesterol in hypertensive rat models [20].

### 5.2. Citrullus colocynthis

Bitter apple possesses potent anticancer activity, especially through cucurbitacin E, which induces cell cycle arrest and apoptosis in HepG2 and MCF-7 cells at concentrations of 5–20 µg/mL [21,40,87,88,89]. Anti-inflammatory effects have been reported via downregulation of TNF-α and IL-6 in animal models of inflammation [32,37,89]. Antibacterial effects have been confirmed against *S. aureus*, *P. aeruginosa*, and *Bacillus subtilis*, with inhibition zones ranging from 15 to 21 mm [34,37,87,88]. The plant also demonstrates antihypertensive activity by modulating nitric oxide synthesis and vasodilation, although clinical validation is limited. It also showed antioxidant potential and exerted activity against some pathogenic fungi (*Candida krusei*, *C. albicans*, *C. parapsilosis*, *C. glabrata*, and *Aspergillus flavus*) [90,91]. Its extract could improve the pathological state of Parkinson’s disease in the MPP+ cell model and the MPTP mouse model [92].

### 5.3. Morus alba

White mulberry exhibits strong anticancer potential, primarily due to flavonoids like morusin and stilbenoids, which inhibit cancer cell proliferation and induce apoptosis in prostate, liver, and breast cancer models [46,93,94]. Its anti-inflammatory action is linked to suppression of NF-κB and MAPK signaling, with significant reductions in TNF-α and IL-1β observed in rodent models [45,94,95,96]. The plant also exhibits broad-spectrum antimicrobial activity against *H. pylori*, *Candida albicans*, and other pathogens [47]. In hypertensive models, extracts of white mulberry have been shown to improve endothelial function and reduce blood pressure through ACE inhibition and antioxidant mechanisms [46,93]. It effectively impedes pseudorabies virus infection by suppressing viral adsorption and entry while also curbing the expression of antiviral cytokines [97]. It also has cardioprotective and neuroprotective activity [93,95].

### 5.4. Rhazya stricta

Harmal-e-shami contains numerous indole alkaloids with established cytotoxic activity against various cancer cell lines, including HeLa and HL-60, with IC_50_ values as low as 2.7 µg/mL [30,53,98,99,100]. Anti-inflammatory activity is attributed to inhibition of 5-lipoxygenase and prostaglandin E2 synthesis [101]. Antimicrobial assays have demonstrated activity against *Klebsiella pneumoniae* and *Staphylococcus aureus*, with inhibition zones reaching up to 19 mm [52]. Additionally, studies report antihypertensive potential via modulation of calcium channel activity and diuretic effects, although clinical data remain scarce [51].

## 6. Conclusions and Future Perspectives

This review consolidates current evidence on the antidiabetic properties of four medicinal plants commonly used in the UAE: caper, bitter apple, white mulberry, and harmal-e-shami. These plants contain a diverse range of bioactive compounds, including flavonoids, alkaloids, cucurbitacins, and polyphenols, that act through various mechanisms, such as α-glucosidase inhibition, insulin sensitization, antioxidant activity, and modulation of metabolic pathways.

Preclinical and clinical studies suggest that caper and white mulberry are particularly promising due to their consistent hypoglycemic effects and favorable safety profiles. bitter apple and harmal-e-shami also show significant potential, although further studies are needed to address variability in efficacy, standardization, and potential toxicity.

Despite the therapeutic potential of these plants, several challenges remain. These include the lack of standardized extracts, a scarcity of large-scale clinical trials, and gaps in understanding their long-term safety and efficacy. Future research should focus on isolating active compounds, clinically validating herbal formulations, and exploring their synergistic effects with conventional antidiabetic drugs.

Preserving and scientifically validating the UAE’s native medicinal flora represents a significant opportunity for developing culturally relevant, plant-based interventions for chronic diseases, such as diabetes. With continued investment in research, these plants may make substantial contributions to sustainable healthcare solutions and the discovery of natural product-based drugs.

## Figures and Tables

**Table 2:** Overview of antidiabetic properties of medicinal plants in the UAE.

**Plant Name**	**Bioactive Compounds**	**Antidiabetic Properties**	**Key References**
Caper	Flavonoids, phenolic acids, alkaloids, volatile oils, fatty acids, and polysaccharides	Improves glucose metabolism, enhances insulin sensitivity, reduces fasting blood glucose, ↓ fasting glucose and HbA1c (400 mg/day in humans), and improved insulin sensitivity (20–400 mg/kg in animal models)	[11,18,20,57,61]
Bitter apple	Cucurbitacins, alkaloids, flavonoids, and phenolic acids	Reduces glycated hemoglobin, improves pancreatic β-cell function, lowers glucose levels, regeneration of β-cells, ↓ glucose (100–300 mg/kg in animals), and ↓ HbA1c in patients (300 mg/day for 2 months)	[21,42,80]
White mulberry	Chlorogenic acid, gentisic acid, flavonoids (rutin and quercetin), and DNJ	Enhances glucose uptake, improves insulin secretion, reduces oxidative stress, ↓ postprandial glucose (1.2 g/day), inhibits α-glucosidase, and enhances insulin sensitivity and glucose uptake	[22,29,38]
Harmal-e-shami	Alkaloids and heterocyclic compounds	Variable outcomes regarding its effects on glucose homeostasis, adiponectin hormone, and insulin regulation; DPP-IV inhibition (up to 61%); ↑ GLP-1 secretion; and ↓ blood glucose and HbA1c (ethyl acetate and methanolic extracts)	[30,51,52,60]

DNJ: 1-deoxynojirimycin; DPP-IV: dipeptidyl peptidase-IV; GLP-1: glucagon-like peptide-1.

## Data Availability

No new data were created or analyzed in this study.
